# Supplementary material for: An Oil-Based Adjuvant Improves Immune Responses Induced by Canine Adenovirus-Vectored Vaccine in Mice
Source: Viruses. 2023 Jul 30;15(8):1664. doi: 10.3390/v15081664 (PMC10458467; doi:10.3390/v15081664)
Supplement: Supplementary file 1 [file viruses-15-01664-s001.zip › viruses-2498821-supplementary.pdf]

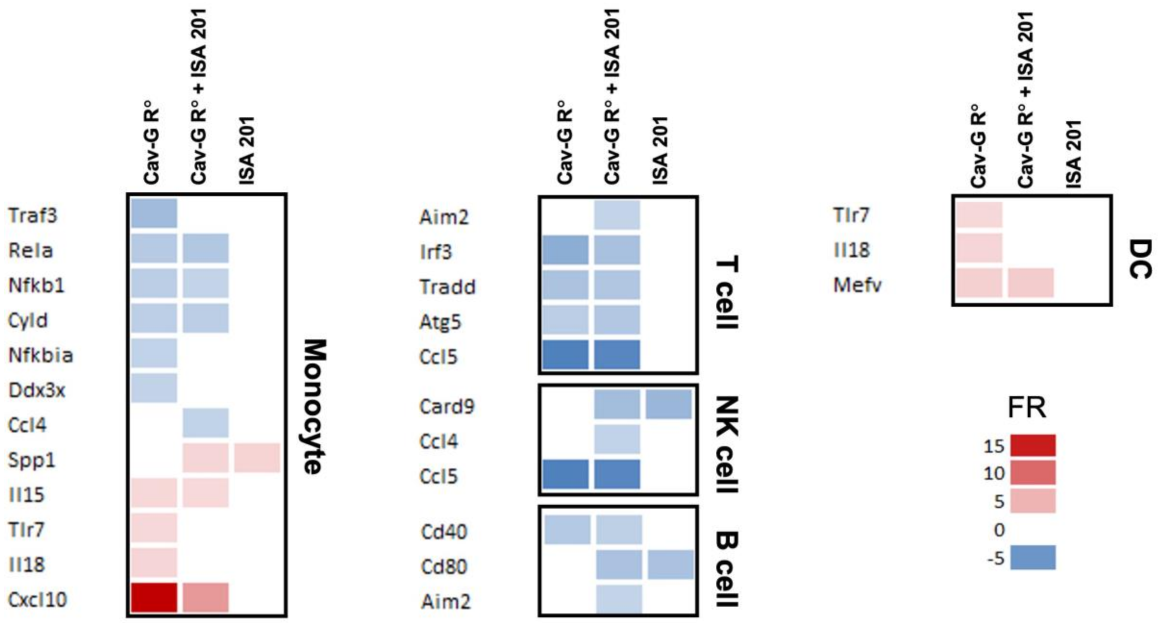

Figure S1. Comparison of antiviral gene modulations in blood, 24 hours after vaccination with Cav-G R^0 and/or ISA 201, using cell-type specific gene lists. The fold regulation (FR) of gene expression was calculated using one pool of whole blood per group of treated mice, with saline solution as the reference. Significant values are represented by red colors, indicating up-regulated gene expression, and blue colors, indicating down-regulated gene expression, in comparison to the mock group. Significantly DEGs were classified by comparing them with cell-type specific gene lists established from human blood [16].
